# Pontine Functional Connectivity Gradients

**DOI:** 10.1007/s12311-025-01943-7

**Published:** 2025-12-20

**Authors:** Paul-Noel Rousseau, Pierre-Louis Bazin, Christopher J. Steele

**Affiliations:** 1 Department of Psychology, Concordia University, Montreal, Canada; 2 Full brain picture Analytics, Leiden, The Netherlands; 3 School of Health, Concordia University, Montreal, Canada; 4 Department of Neurology, Max Planck Institute for Human Cognitive and Brain Sciences, Leipzig, Germany

**Keywords:** Cerebellum, Pons, Pontine nuclei, Connectivity, Gradients

## Abstract

The cerebellum contributes to a wide range of motor and non-motor functions through its extensive connections with much of the cerebral cortex. The pons plays a key role in these connections. It receives inputs from much of the cerebral cortex, and in turn projects to cerebellar cortex. The anatomical organization of corticopontine and pontocerebellar connectivity has been well described, but the functional organization of the pons in humans remains poorly understood. In particular, it is unclear how inputs from different areas are integrated or segregated within it. We sought to characterize the functional organization of the pons based on its functional connectivity with the cerebral and cerebellar cortices. To this end, we used diffusion map embedding, a non-linear dimensionality reduction technique, to reconstruct gradients that reflect the dominant patterns of pontine functional connectivity. Our results reveal a primary gradient in the pons that is organized along rostrocaudal and mediolateral axes. We found that inputs from higher-order cognitive areas of the cerebral cortex converge on rostral and medial pons, whereas inputs from sensorimotor areas converge on the caudal and lateral pons. The organization of this gradient mirrors the unimodal-to-transmodal gradient previously described in the cerebral and cerebellar cortices. The observed patterns align with findings from invasive tract-tracing in non-human animals and extend them to demonstrate that pontine functional organization can be characterized by a complex pattern of segregation and integration. These findings highlight that, rather than serving as a simple relay, the pons may function as an integrative hub within the cortico-ponto-cerebellar pathway.

**Supplementary Information:**

The online version contains supplementary material available at 10.1007/s12311-025-01943-7.

## Introduction

The cerebellum is increasingly recognized as contributing to virtually every facet of cognition and behaviour (Sokolov et al. 2017). The uniform cellular architecture of the cerebellar cortex suggests that its contributions are shaped by precisely organized reciprocal connections with the cerebral cortex and the rest of the brain (Schmahmann et al. 2019). Understanding the organization of corticocerebellar loops is therefore essential to furthering our understanding of the function of the cerebellum. Bridging the downstream connections between the cerebral cortex and the cerebellum are the pontine nuclei of the pons (Schmahmann et al. 2019). The organization of corticopontine and pontocerebellar connections has been characterized over decades of invasive animal research (e.g.Brodal 1979; Glickstein et al. 1985; Schmahmann and Pandya 1997b). More recently, key findings from the animal literature demonstrating topographic organizational principles within these connections have been recapitulated with non-invasive diffusion MRI tractography studies in humans (Rousseau et al. 2022, 2025). Despite these recent insights, our knowledge of how the pathway is organized in humans and of its role in transmitting, and potentially transforming (Schwarz and Thier 1999) cerebrocortical information destined for the cerebellum remains limited.

**Fig. 1:**
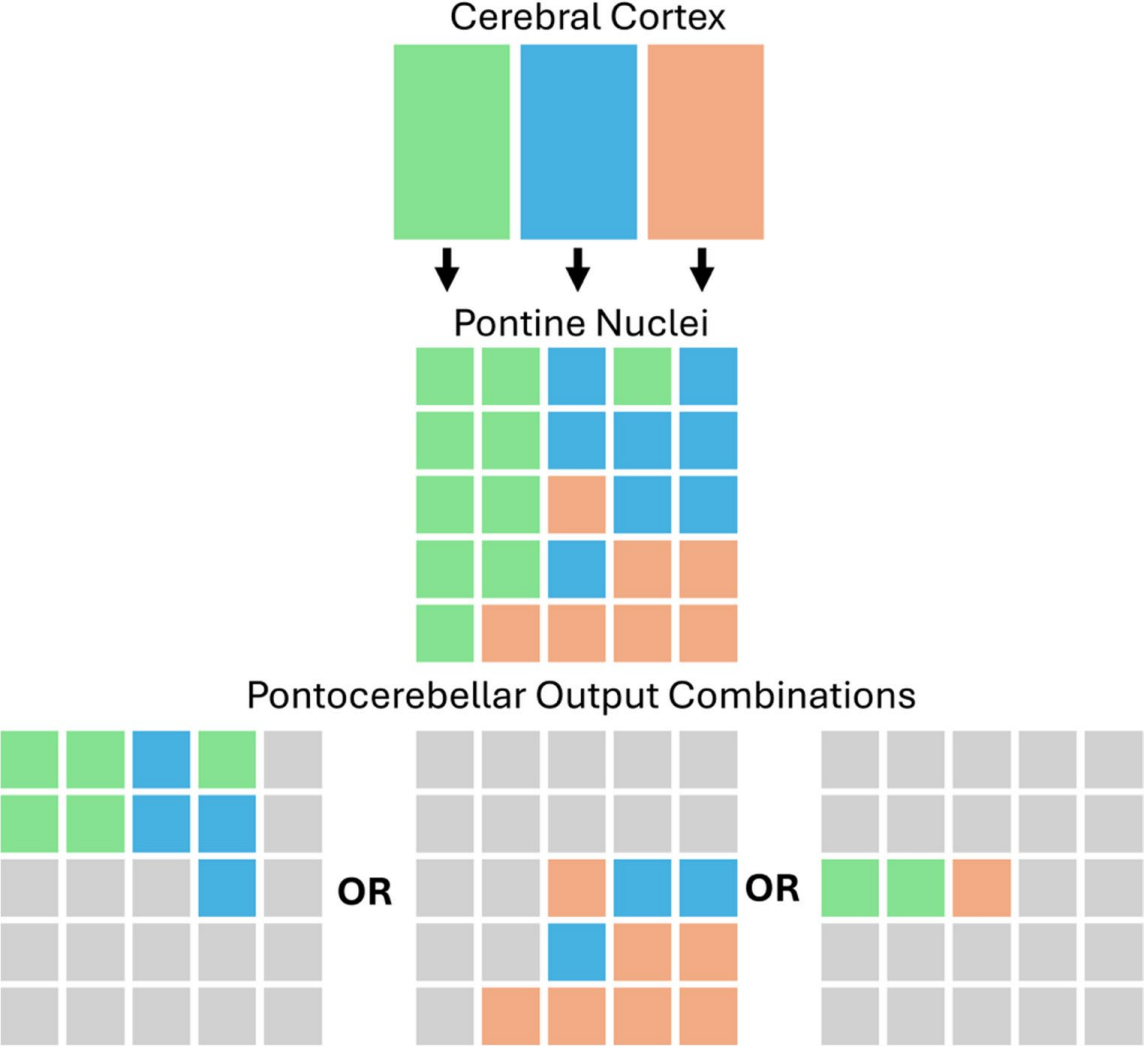
Schematic, adapted from Schwarz and Thier (1999), illustrating the pons as selectively integrating and relaying particular combinations of corticopontine inputs to the cerebellum. The cerebral cortex is depicted as a two-dimensional sheet, subdivided into three spatially distinct regions (colored green, blue, and orange), that project to the pons in a patchy mosaic pattern. Cortical topography is partially preserved, but with interdigitation of inputs from spatially distinct cortical sites. Specific combinations of adjacent corticopontine inputs may then be selectively relayed to the cerebellum

Much of the cerebral cortex contributes to the estimated 40 million axons forming the corticopontine projection, one of the most substantial projection systems in the brain (Leergaard and Bjaalie 2007; Schwarz and Thier 1999). Corticopontine terminals are organized in a complex topographic manner, with different cerebrocortical sites projecting to demarcated patches within the pons (Schmahmann and Pandya 1997b). The projection is characterized by both convergence – distant cortical areas convergence on nearby pontine patches – and divergence whereby a discrete cortical area may project to diffusely distributed patches in the pons (Brodal 1978). While the spatial relationships between different cortical areas are partially preserved, the distinctive arrangement of corticopontine terminals results in an interdigitation of patches from disparate cortical areas (Schmahmann and Pandya 1997b). This anatomical configuration – which brings together inputs from different cerebrocortical sites along with filter-like electrophysiological characteristics of pontine neurons – has been proposed to enable the integration of different combinations of inputs before they are relayed to the cerebellum (depicted in Fig. 1) (Schwarz and Thier 1999). Approximately half as many fibres that enter the pons exit as mossy fibres that target the cerebellar cortex in a complex convergent and divergent manner (Biswas et al. 2019; Kratochwil et al. 2017; Schwarz and Thier 1999). Compared with the pons, the cerebellar cortex is ill suited to integrating distributed inputs: its circuitry is organized in a modular fashion with a limited capacity for coordinating signals across its surface (Schwarz and Thier 1999). Cerebellar cortical outputs are conveyed via the deep cerebellar nuclei to the thalamus and then back to the cerebral cortex in a topographically organized manner, thereby creating a closed loop (Dum and Strick 2003). Overall, the cortico-ponto-cerebellar segment of the circuit is characterized by the partial preservation of the cerebral cortex’s topography, but with the pons serving as a possible site for the integration or recombination of different cerebrocortical inputs.

Two widely used methods for studying connectivity in humans are diffusion MRI (dMRI) tractography and resting state functional MRI. dMRI investigations (e.g., Rousseau et al. 2022, 2025) have informed understanding of different portions of the cortico-ponto-cerebellar pathway in humans, but they have important limitations. First, dMRI tractography cannot accurately resolve multi-synaptic pathways and therefore cannot capture the organization of the entire circuit (Lundell and Steele 2024). Second, while dMRI can reveal macroscale organization of afferent and efferent pontine connections, it does not contribute to our understanding of the pons as a functionally dynamic structure – that is, how different inputs from different areas of the cortex may be integrated within it and selectively relayed to the cerebellum. Integration in this sense is proposed to be both spatial and temporal, with spatial integration reflecting the convergence of inputs from different cortical areas/networks, and temporal integration reflecting the selective gating or timing of specific inputs as they are relayed to the cerebellum. Functional connectivity based on resting state functional MRI provides complementary and novel perspectives to prior anatomical and dMRI studies (Buckner et al. 2011).

Functional connectivity reveals organization across the multiple levels of the cortico-ponto-cerebellar pathway. Furthermore, it enables investigation of how distinct cerebrocortical areas/networks – and by extension cognitive functions – are represented within the pons. Seed based functional connectivity approaches have demonstrated that different cerebrocortical areas connect to the pons in a topographically organized manner, resembling patterns observed in non-human primate work (Karbasforoushan et al. 2022). An alternative, data-driven method is functional connectivity gradients, which have been used to map fundamental principles of cerebral (Margulies et al. 2016) and cerebellar cortical (Guell et al. 2018) functional organization. This approach allows researchers to derive mappings of continuous transitions in connectivity signatures across the brain, which are expressed as gradients. Gradients are anchored by two poles, where each pole represents different anchor points characterized by distinct connectivity profiles with a transitional area that blends between them. Given the proposed dynamic functional architecture in the pons (Schwarz and Thier 1999), the gradients approach is well suited to disentangling overlapping organizational principles identifying distinct connectivity patterns with the cerebral and cerebellar cortices.

In the present study, we aimed to uncover the principles of functional organization of the pons to better characterize its role in the corticocerebellar pathway. To this end, we reconstructed functional connectivity gradients within the pons based on its connectivity from the cerebral cortex and to the cerebellar cortex in a large resting state functional magnetic resonance imaging dataset of 1003 individuals from the Human Connectome Project (Van Essen et al. 2013). Given the well documented challenges of fMRI in the brainstem (e.g., Beissner 2015), the averaging of a large number of datasets available from the HCP is required to improve the signal to noise ratio (SNR) and allow a reliable delineation of connectivity. We projected the principal pontine gradient to its input/output structures (cerebral and cerebellar cortices) to map their functional correspondence and contextualize the putative integrative role of the pons. The present study advances our understanding of organization of the human cortico-ponto-cerebellar pathway and contributes to evidence that the pons is not a simple relay but serves to integrate information from disparate cortical areas.

## Materials and Methods

### Connectivity Dataset

The Human Connectome Project’s (Van Essen et al. 2013) dense connectome dataset, downloaded from Connectome-DB (https://db.humanconnectome.org), was used for all analyses in the present study. Very briefly, this is a correlation matrix that represents the average correlation between all pairs of brain coordinates (cerebral cortical vertices and subcortical voxels, matched across individuals) for 1003 individuals from the HCP’s s1200 release (WU-Minn HCP Consortium 2017). The raw data was based on 1-hour of resting state fMRI data that were concatenated across four fMRI runs for each of the individual participants. The data collection and preprocessing parameters are described in detail by (Glasser et al. 2016). ((TR) of 720 ms, echo time (TE) of 33.1 ms, and 2 mm isotropic resolution). The procedure used to generate the dense connectome is detailed in HCP Documentation (Human Connectome Project 2017).

### Definition of the Cortico-Ponto-Cerebellar Connectivity Matrix

Using HCP’s Workbench Command (https://www.humanconnectome.org/software/workbench-command), we constrained the whole brain correlation matrix to include only the pons, cerebral cortex, and cerebellar cortex. While the cerebral cortical coordinates were already defined, we utilized a hand-drawn mask of the pons, and a mask of the cerebellar cortex based on FSL’s cerebellar atlas (Diedrichsen et al. 2009) as regions of interest to identify appropriate voxels. The final connectivity matrix represents the connectivity between 1213 pons grayordinates, and grayordinates in the cerebral cortex (59412) and cerebellar cortex (17709). The aim was to capture the connectivity between the pons and its primary input and output structures. Note that we explicitly did not include intrinsic pontine or cerebellar cortical connections, nor did we include cerebellar to cerebral cortex connections. While the pons is integrated within larger subcortical and brainstem networks, we focused on connections from the cerebral cortex and to the cerebellar cortex, both because they represent the largest pontine inputs and outputs and to facilitate comparisons with prior dMRI tractography and anatomical studies. Intrinsic pontine connections were specifically excluded for this same reason, as our goal was to characterize the dominant input and output pathways. This final connectivity matrix was used as the basis for all subsequent analyses.

### Gradient Extraction with Diffusion Map Embedding

Diffusion map embedding was used to identify five gradients within the pons based on its connectivity with the cerebral and cerebellar cortices. As described above, intrinsic pontine, cerebrocortical, and cerebellocortical connectivity were not considered in the analysis. Analyses were performed using procedures and code adapted from Guell et al. (2018) and available at https://github.com/xaviergp/cerebellum_gradients, which implements diffusion map embedding with the mapalign package (https://github.com/sensein/mapalign). Diffusion map embedding is a non-linear dimensionality reduction technique initially introduced by Coifman and Lafon (2006) and applied to human connectivity data in a seminal study by Margulies et al. (2016). Diffusion map embedding allows for the representation of high-dimensional connectivity data, in our case the connectivity between 1213 pons nodes and 77,121 cerebral and cerebellar cortical nodes, in a lower dimension space that encapsulates the dominant patterns or gradients of connectivity. Prior to diffusion map embedding, the correlation matrix was transformed into an affinity matrix using the cosine similarity metric, following previous studies (Margulies et al. 2016; Guell et al. 2018). This approach facilitated direct comparisons between our results and those prior findings. The affinity matrix effectively represents the similarity of connectivity between pairs of pontine nodes. Diffusion map embedding was then performed on the affinity matrix to extract the modes (or components) that represent the connectivity gradients within the pons (ordered by decreasing amount of variance accounted for).

### Projection of Pontine Gradients to the Cerebral and Cerebellar Cortices

In order to investigate the correspondence between pontine gradients and connectivity with the cerebral and cerebellar cortices we then “project” pontine gradients by performing the dot product between the embedding values within the pons (a 1212 by 1 array) and the pons by cerebellar cortex and pons by cerebellar cortex connectivity matrices (1213 x N nodes arrays) separately as in previous work (e.g., Guell et al. 2020; Katsumi et al. 2023). Each node in the cerebral cortex and cerebellar cortex thereby receives a value representing the relative strength of its connectivity to the pontine voxels, weighted by pontine gradient values. Cerebral cortical projection maps were visualized using Connectome Workbench (Marcus et al. 2011). Cerebellar projection maps were first converted from volumetric space to cerebellar surface-based flatmaps using SUIT (Diedrichsen 2006; Diedrichsen and Zotow 2015) and then visualized with Connectome Workbench.

### Residuals Analysis

We conducted a post-hoc residuals analysis to directly compare our cerebrocortical and cerebellar cortical gradient projections with the published gradients of Margulies (2016) and Guell et al. (2018). This approach was designed as a way to visualize and quantify systematic regional differences between these gradients and our gradient projections. These residuals reflect shifts along the unimodal-transmodal continuum in our gradient projections relative to the previously published gradients. In other words, it informs us which regions are more unimodal, transmodal or intermediate in our gradient projections in comparison with these canonical gradients. In each case, a linear regression was performed with the previously published gradient being predicted by our corresponding projection. Residuals were plotted either on the cerebrocortical surface, or on a volume of cerebellar cortex that was converted to a cerebellar flatmap using SUIT (Diedrichsen 2006; Diedrichsen and Zotow 2015). All results were visualized with Connectome Workbench.

## Results

### Pontine Connectivity Gradients

**Fig. 2:**
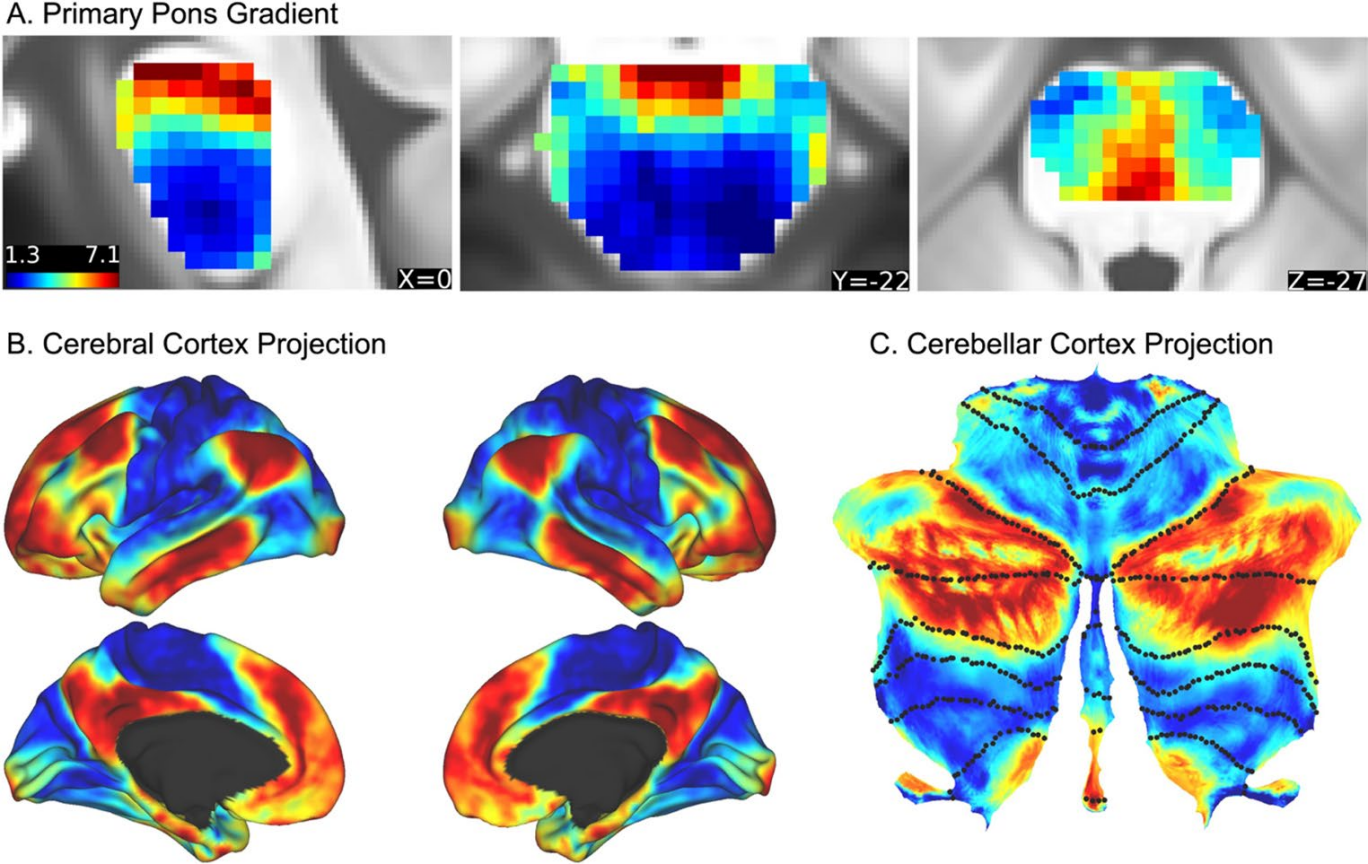
Pontine connectivity gradient and corresponding cerebral and cerebellar cortical projections. **(A)** The primary gradient in the pons based on its connectivity to cerebral and cerebellar cortices, shown on sagittal, coronal, and axial slices (x = 0, y = − 22, z = −27). **(B)** The cerebral cortex projection of the primary gradient. **(C)** The cerebellar cortical projection of the primary gradient, depicted on a cerebellar flatmap generated with SUIT tools (Diedrichsen and Zotow 2015)

The first gradient, depicted in Fig. 2a, explained over 70% of the variability in cortico-ponto-cerebellar functional connectivity (see Supplementary Fig. 1 for a graph of variance accounted for by each gradient). Similarly coloured voxels exhibit similar patterns of functional connectivity jointly with the cerebral cortex and the cerebellum. We observed two directions of transition within the three-dimensional structure of the pons, reflecting distinct streams of cerebrocortical and cerebellocortical connectivity. First, there is a transition along the rostral-to-caudal axis. Second, in the rostral pons we identified a medial-to-lateral transition. The gradient shows a sharp transition just below the rostral third of the pons, beyond which it remains largely uniform throughout the caudal pons.

When this gradient was projected to the cerebral cortex (see Fig. 2b), we observe a pattern that recapitulates the unimodal-to-transmodal organization previously described there (Margulies et al. 2016). The rostral/medial pons shows stronger weighted connectivity to higher-order, association areas, notably areas of the default mode network (DMN) (depicted in warm colours). Caudal/lateral pons corresponds to primary and secondary sensorimotor areas including primary motor cortex, pre-motor and cortex and supplementary motor areas, auditory cortex, and secondary visual areas (depicted in cool colours). In addition to primary and secondary sensorimotor areas, we also see correspondence with dorsal and ventral attention network areas. The cerebellar projection (Fig. 2c) roughly corresponds to a non-motor versus motor hierarchical functional organization previously demonstrated in resting-state and task-based work (Buckner et al. 2011; King et al. 2019). The rostral/medial pons shows stronger weighted connectivity to Crus I and II and lobule IX (warm colours), whereas lateral/caudal pons corresponds more to lobules I-VI and VIIB-VIIIB (cool colours).

Gradients 2 and 3 (see Supplementary Fig. 2) appear be dominated by laterality effects, with sharp transitions of embedding values at the midline of the pons. These likely reflect the predominantly ipsilateral projections from the cerebral cortex to the pons, and contralateral connections from pons to cerebellum. Gradient 4 captures a medial-lateral axis that separates motor and cognitive control cerebrocortical areas from visual areas. Gradient 5 is organized along a dorsal-ventral axis, that reflects a segregation of sensory/cingulate cortical areas and frontal/temporal association areas. Given the lateral effects in gradients 2–3, and the lower variance accounted by 4–5 (less than 5% each), these are described here for completeness, but we focused our primary analysis on the principal gradient.

### Residuals Analysis

**Fig. 3:**
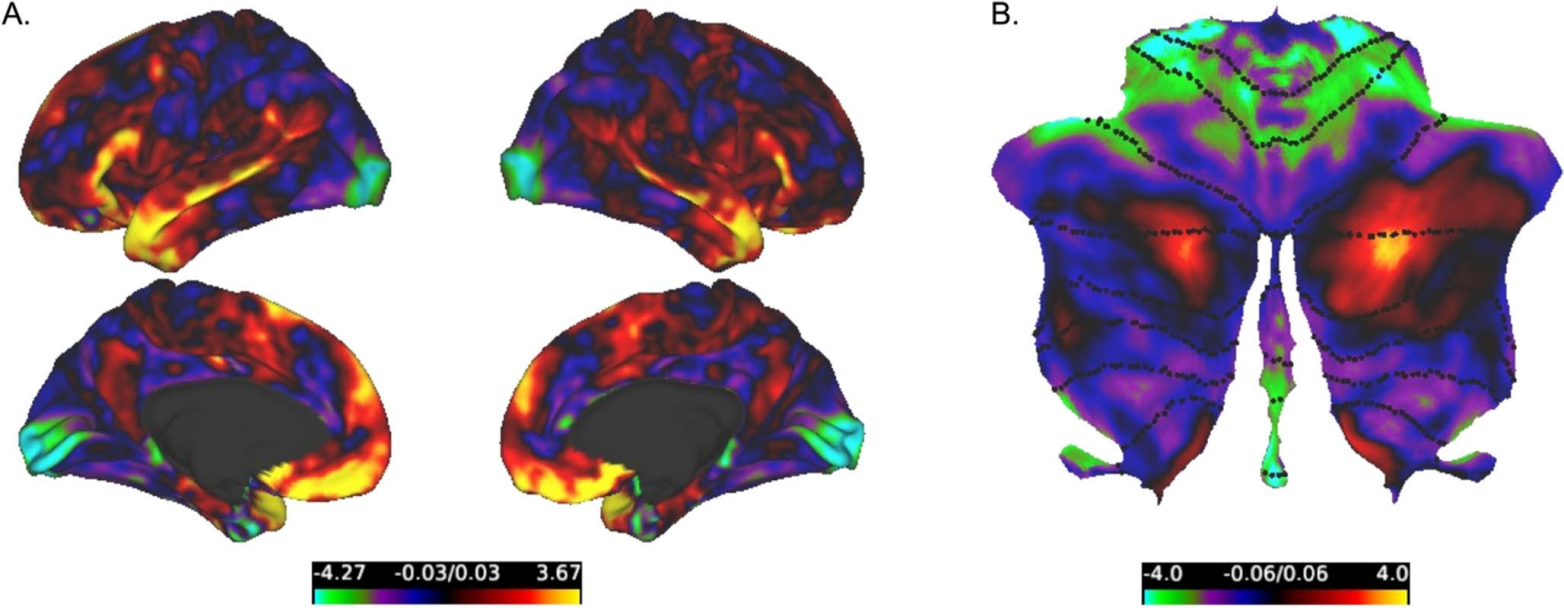
Residual analysis comparing our gradient projections to previously published cerebrocortical and cerebellar gradients. **(A)** Residuals between our cerebrocortical gradient projection and the principal gradient of Margulies (2016). **(B)** Residuals between our cerebellar cortical projection and the principal gradient of Guell et al. (2018). Positive residuals indicate regions where the referenced gradients show higher gradient values compared to ours; negative residuals indicate regions where our gradients projections show higher values

Results of the residuals analysis are presented in Fig. 3. Residual values reflect shifts in the organization of our gradient projections relative to those of Margulies (2016) and Guell et al. (2018), both of which are interpreted as reflecting a unimodal-transmodal organization. Negative residuals indicate a shift in our projection toward the transmodal end of the gradient (i.e., DMN), whereas positive residuals indicate a shift towards the unimodal end (i.e., sensorimotor areas). In the cerebral cortex (Fig. 3a), we identified the most prominent negative residuals in areas of visual cortex, and positive residuals in inferior frontal gyrus, middle temporal gyrus, temporal pole, and medial prefrontal cortex. In the cerebellar cortex (Fig. 3b) we identified the post prominent negative residuals in areas of lobules IV-VI, vermal portions of VIIIB and IX, and positive residuals in in areas of Crus I and Crus II and hemispheric portions of IX. Residuals appear to show some lateralization effects, in that we identified higher positive residuals in left inferior frontal gyrus, and right Crus I and Crus II.

## Discussion

We reconstructed the functional connectivity gradients of the pons based on its connectivity with the cerebral and cerebellar cortices to uncover principles governing its functional organization. We found that the primary functional gradient of the pons was organized in a rostral-to-caudal and medial-to-lateral fashion that, when projected onto the cerebral and cerebellar cortices, formed gradients that corresponded closely with the unimodal-to-transmodal organization seen in prior work (Guell et al. 2018; Margulies et al. 2016). Rostral and medial areas of the pons were preferentially connected to default mode and frontoparietal network areas, whereas caudal pons aligned with somatomotor, dorsal and ventral attention network areas of the cerebral and cerebellar cortices. Our results align closely with previous gradient work, but residuals indicated subtle but systematic differences localized in specific cortical and cerebellar areas suggesting overlapping network represents within particular areas of the pons. Together, these findings provide supporting evidence that the pons has a complex functional organization and may serve as an integrative hub in the downstream corticocerebellar pathway.

A rostral-caudal and medial-lateral functional connectivity gradient in the pons is remarkably consistent with anatomical connectivity work in non-human primates (Schmahmann et al. 2004; Schmahmann and Pandya 1997b) and our recent dMRI tractography work in humans (Rousseau et al. 2022, 2025). Specifically, motor projections to the pons have been shown to terminate preferentially in the caudal half of the pons (Schmahmann et al. 2004), which is also the origin of pontocerebellar fibres projecting to cerebellar motor areas (Brodal 1979; Rousseau et al. 2022). Prefrontal cerebrocortical areas target the medial pons (Schmahmann and Pandya 1997a). Medial prefrontal cortex targets rostral pons whereas dorsolateral prefrontal projections are distributed along its rostral-caudal extent (Schmahmann and Pandya 1997a). Different areas of the parietal cortex also show differential patterns of projection to the lateral pons, with the inferior parietal lobule projecting rostrally and the superior parietal lobule projecting more diffusely along the rostro-caudal extent (Schmahmann and Pandya 1989). These anatomical findings are mirrored in our primary gradient, which shows sensorimotor areas clearly localized to the caudal pons and a more complex, and distributed pattern for non-sensorimotor regions. Specifically, medial prefrontal areas and the inferior parietal lobule – both nodes in the default mode network – correspond to the medial and rostral portions of the pons in our gradient. Conversely, the superior parietal lobule and portions of dorsolateral prefrontal cortex show a wider distribution of connectivity with the caudal two thirds of the pons. Broadly, our findings correspond with organizational patterns demonstrated in the anatomical literature.

While our findings align with certain aspects of known anatomical connectivity, they diverge in ways that highlight the complexity of comparing structural and functional connectivity. For instance, detailed work in macaques has demonstrated that temporal lobe corticopontine projections arise predominantly from the upper bank of the superior temporal sulcus and the superior temporal gyrus (Schmahmann and Pandya 1991). However, in our gradient, we found that the rostral pons maps onto large areas of middle and inferior temporal gyri. Without excluding the possibility that the anatomical connectivity is different in humans, this is more likely to reflect indirect connections mediated at the level of the cerebral cortex (Damoiseaux and Greicius 2009). These temporal regions are highly interconnected with other cortical areas with known projections to the pons in non-human primates (e.g., inferior parietal lobule). It follows that the functional connectivity of the pons, and by extension our gradient, is shaped by a combination of direct and indirect cortical inputs. In addition, though we excluded pons-pons connectivity from our analysis we cannot rule out potential indirect contributions that could be mediated by pontine interneurons (Mihailoff et al. 1992) and/or projections from the deep cerebellar nuclei (Schwarz and Thier 1999).

Our findings also differ from those of Margulies (2016) and Guell et al. (2018) in a manner that may suggest a possible integrative function in the pons. In our residual analysis, when comparing our gradient projections with the primary gradient of Margulies (2016), we found strong negative residuals in visual cortex. This finding suggests that (relative to the canonical gradient presented by Margulies (2016) visual areas in our gradient projection are situated towards the middle of the gradient, whereas in Margulies (2016) these same areas clearly correspond to the unimodal end of their primary gradient. The anatomical tract-tracing work provides compelling evidence that ventral stream visual areas project along the rostro-caudal extent of the pons (Schmahmann and Pandya 1993). With our dataset, connectivity from visual areas to the pons do not appear to be spatially specific, in line with these anatomical findings. This results in the projection to this area sampling values from both ends of the pontine gradient, leading to more intermediate values. By extension, this also implies that visual inputs to the pons are in proximity to both motor inputs and inputs from associative cortical areas, thus providing the spatial proximity necessary for integration (Schwarz and Thier 1999). In our residual analyses, we also found strong positive residuals in the inferior frontal gyrus, middle temporal gyrus, and regions of the cerebellar cortex that are recruited during language production tasks (King et al. 2019). This pattern suggests that, in our gradient projection, these language related regions are shifted towards the unimodal end of the functional hierarchy relative to the cerebrocortical and cerebellar cortical gradients of Margulies (2016) and Guell et al. (2018). It may imply that at the level of the pons, language and sensorimotor channels may be more integrated or overlapping than in the cerebral or cerebellar cortices.

The anatomical tracing work shows that corticopontine terminations from different cerebrocortical areas are interdigitated with one another, bringing inputs from different cerebrocortical areas (e.g., visual and motor) in close proximity to one another. Schwarz and Thier (1999) propose that this arrangement complements filter-like characteristics of pontocerebellar neurons to allow the pons to integrate signals from different regions before projection to the cerebellum as mossy fibres. They suggest that depending on context or specific environmental demands, different combinations of corticopontine inputs can be integrated and relayed to the cerebellum (see Fig. 1). Our findings align with and support portions of this theory by demonstrating the convergence of different functionally relevant inputs to overlapping zones within the pons. Thus, our results provide evidence that visual information may be integrated with motor related signals (e.g., for visually guided reaching) or coupled with non-motor signals (e.g., for non-verbal working memory) at the level of the pons. Similarly, language-related inputs may be integrated with sensorimotor information or with signals from multimodal association areas depending on contextual demands.

The fact that cortical, pontine, and cerebellar nodes of the cortico-ponto-cerebellar pathway appear to be organized in a similar hierarchical fashion may be interpreted as the cortex imprinting its functional architecture on downstream areas. Alternatively there may be more of a reciprocal relationship such that the pons and cerebellum may together influence integration at the level of the cortex. Developmentally, the spatial organization of pontine neurons is established early, and these neurons provide cues that guide the termination patterns of corticospinal axons (Kratochwil et al. 2017). This suggests that the pons actively reshapes the configuration of cerebrocortical inputs, rather than merely reflecting their organization. The gradients observed in the present study likely reflect a combination of macroscale organizational principles, possibly reflecting the anatomical configuration of these pathways, and influences of experience and cerebellar learning. Schwarz and Their (1999) emphasize the role of projections from the deep cerebellar nuclei to the pons in shaping the information it relays to the cerebellum. Within this framework output from cerebellar nuclei – which is shaped by cerebellar models and learning – plays a role in sculpting the functional organization of the pons. It follows that experiences and pathological factors may influence the organization of pontine gradients, highlighting an important direction for future work.

While the present findings provide a biologically plausible characterization of pontine functional organization, they must also be interpreted within the context of brainstem specific methodological limitations. We cannot rule out that the observed patterns of convergence of functionally distinct inputs in the pons are influenced by the small size of this structure and the level of spatial resolution of the dataset. We also note that relative to prior gradient work (e.g., Guell et al. 2020; Katsumi et al. 2023; Margulies et al. 2016) with the same or similar sample size, the cerebral cortex and cerebellar cortical projection gradients presented here exhibit more “noise”. This can be appreciated as a relative decrease in spatial smoothness across the gradient projections. Even with this large sample of high-quality fMRI data used in the present study, we still face important limitations related to low SNR in the pons that represent a barrier to more granular investigations of its functional topography. For instance, while there may be important information within the other gradients, the potential influence of noise and prominent laterality effects led us to focus our interpretation on the first gradient, which we are confident reflects a robust and meaningful pattern. The brainstem represents a worst-case scenario for functional MRI: it is compact in size with densely packed and functionally distinct nuclei, there is poor differentiation of gray matter nuclei from surrounding white matter at the resolutions possible with in-vivo MRI, and there is both elevated physiological noise and magnetic susceptibility artefacts (Sclocco et al. 2018). Group registration may also lead to misalignment of smaller brainstem nuclei, which serves to further reduce the effective resolution of fMRI (Sclocco et al. 2018). Some of these limitations are partially mitigated by averaging a large number of high qualities rsfMRI datasets, as was also done in the present study. However, this approach may also affect the level of granularity with which we can investigate the pons.

Moving forward, replication of the present findings at higher spatial resolutions using acquisitions more tailored to the brainstem is needed. Improved brainstem data quality would also allow for the extraction of meaningful gradients at the individual participant level. This represents an important goal for future work, as it would enable an examination of individual variability and different experiential or pathological factors shaping pontine gradients. Future fMRI studies of the pons would benefit from the collection of physiological recordings, use of models of the hemodynamic response function tailored to the brainstem, probabilistic atlases of brainstem nuclei that can improve inter-subject registration, and the use of high-field (e.g., 7 T) MRI data (Groot et al. 2024; Sclocco et al. 2018). Mohamed and colleagues (2024), for instance, developed a specific protocol for brainstem fMRI with 3 T and demonstrated significant improvements in SNR compared to an HCP style protocol. Improved brainstem data quality will lend itself to understanding the spatial distribution of pontine activation in task-based paradigms (e.g., similar to the approach of King et al. (2019) for the cerebellum), allow for the further delineation of pontine resting connectivity gradients and help clarify how these patterns vary across individuals.

## Conclusion

We reconstructed the primary functional gradient in the pons based on its connectivity with cerebral and cerebellar cortices. Our analysis demonstrated that the primary gradient is organized in a rostrocaudal and mediolateral fashion that is consistent with non-human primate tract tracing and dMRI studies in humans (Rousseau et al. 2022, 2025; Schmahmann et al. 2004; Schmahmann and Pandya 1997b). When projected to the cerebral and cerebellar cortex, the pontine-derived gradient aligns closely with the primary gradients observed within each of these separately (Guell et al. 2018; Margulies et al. 2016) but with important regional differences pointing to a level of reorganization of cerebrocortical information in the pons. Our findings provide initial in-vivo human evidence for functional integration in the pons that suggests that the pons may bind functionally relevant inputs from different cerbrocortical areas. Together our results highlight the pons as a dynamic and integrative structure that plays a complex role in the cortico-ponto-cerebellar pathway. These findings add impetus to further investigation into the nature of its contributions to information integration and transformation along this pathway.

## Supplementary Information

Below is the link to the electronic supplementary material.

Supplementary Material 1 (DOCX 1.19 MB)

## Data Availability

The imaging data used in this study were obtained from the Human Connectome Project (https://db.humanconnectome.org/). The code adapted for this study, along with the resulting gradient maps, will be made available at https://github.com/neuralabc.
