# Supplementary material for: Pontine Functional Connectivity Gradients
Source: Cerebellum. 2025 Dec 20;25(1):1. doi: 10.1007/s12311-025-01943-7 (PMC12718233; doi:10.1007/s12311-025-01943-7)
Supplement: Supplementary file 1 — Supplementary Material 1 (DOCX 1.19 MB) [file 12311_2025_1943_MOESM1_ESM.docx]

## **Supplementary Information**


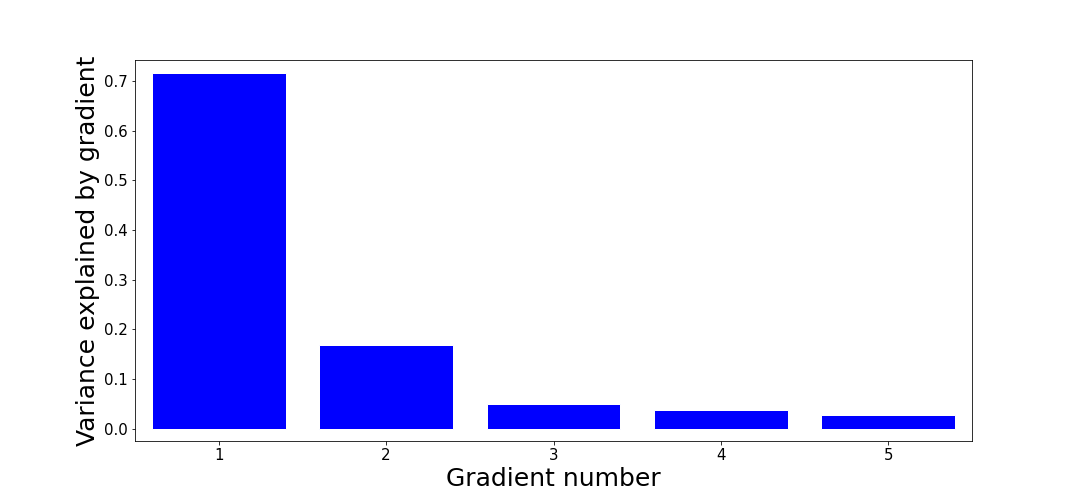


**Supplementary Fig. 1** Proportion variance accounted for by the five reconstructed gradients in the pons. Gradient 1 accounts for approximately 70% of the variance in the connectivity data.


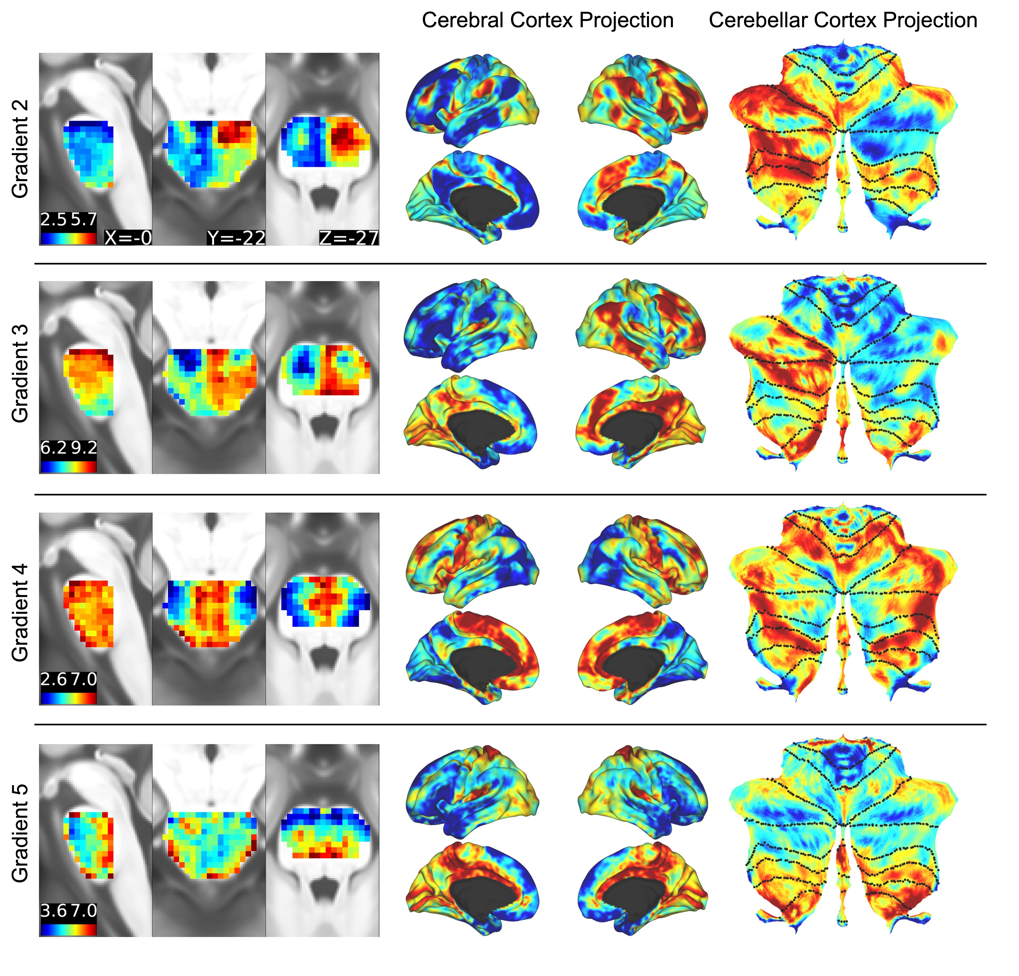


**Supplementary Fig. 2** Pontine gradients 2-5 are depicted on the left. The cerebral cortex projection for each of the gradients is depicted in the centre, and the cerebellar cortical projection (displayed on a cerebellar cortical flat map) is depicted on the right.
